# Area-level associations of travel behaviour metrics with waist circumference: findings from linkage of travel and health surveys

**DOI:** 10.1038/s41598-023-35335-w

**Published:** 2023-05-19

**Authors:** Takemi Sugiyama, Rachel Cole, Nyssa Hadgraft, Neville Owen, Russel G. Thompson, Manoj Chandrabose

**Affiliations:** 1 Centre for Urban Transitions, Swinburne University of Technology, Melbourne, John Street, Hawthorn, VIC 3122 Australia; 2 Baker Heart and Diabetes Institute, Melbourne, Australia; 3 School of Health and Behavioural Sciences, University of the Sunshine Coast, Sippy Downs, Australia; 4 Department of Infrastructure Engineering, The University of Melbourne, Melbourne, Australia

**Keywords:** Public health, Preventive medicine

## Abstract

Individual-level analyses have consistently shown associations of travel behaviours with obesity-related measures. However, transport planning policies often target areas rather than individuals. To better inform transport-related policies and initiatives for obesity prevention, area-level relationships need to be investigated. This study linked data from two travel surveys with data from the Australian National Health Survey at the level of Population Health Areas (PHAs) and examined to what extent area-level travel behaviours metrics (prevalence of active travel, mixed travel and sedentary travel, diversity of travel modes) were associated with the rate of high waist circumference. Data from 51,987 travel survey participants were aggregated into 327 PHAs. Bayesian conditional autoregressive models were used to account for spatial autocorrelation. It was found that statistically replacing participants who relied on cars for travel (without walking/cycling) with those engaging in 30+ min/d of walking/cycling (without car use) was associated with a lower rate of high waist circumference. Areas with greater diversity of travel modes (mix of walking/cycling, car use, public transport use) also had lower prevalence of high waist circumference. This data-linkage study suggests that area-level transport planning strategies addressing car dependency, shifting car use to walking/cycling over 30 min/d, may help to reduce obesity.

## Introduction

The global burden of overweight and obesity is increasing worldwide^1^. The prevalence of overweight/obesity in Australian adults was 67% in 2017–18, and the corresponding rates are similarly high in other Western countries^2,3^. Given the established associations of higher amounts of physical activity and lower amounts of time spent sitting with lower levels of obesity-related measures^4–7^, population-based approaches to increasing physical activity and reducing sedentary behaviour are identified as key strategies for preventing obesity and enhancing public health^8^.

Transport is a relevant domain in this context. Research has found consistent protective associations of active travel (walking and cycling) with obesity measures^9–12^. It has been also shown that longer sedentary travel (time spent sitting in cars) is linked to a higher risk of obesity^13,14^. Since these travel behaviours are conducted on a regular basis by large numbers of people who commute and go out for errands, increasing active travel and decreasing sedentary travel are likely to help to reduce the population health burden of obesity.

Studies on travel behaviours and overweight/obesity have typically examined the relationships at the individual level^15^. Although such individual-level investigations provide the evidence supporting the health benefits of active travel, it should be noted that transport planning policies, particularly those dealing with infrastructure, are generally targeted and implemented at the area level rather than the individual level^16^. Ecological studies examining area-level relationships between travel behaviours and obesity can further inform transport-related policies and initiatives for obesity prevention by supplementing findings from existing individual-level studies.

A challenge in conducting such ecological studies is the lack of data that contain both travel behaviour and health measures at an area level^17^. Although there are datasets that include these measures at the level of individuals, they often collect data from a limited geographic context, which makes it difficult to obtain robust findings due to a smaller number of area units. An alternative approach is to link travel and health surveys, conducted separately by transport and health agencies, using an area as the unit for linkage. Household travel surveys and population health surveys are both publicly available datasets with large samples recruited from a large number of diverse geographic areas.

There are a few data-linkage studies using travel and health surveys that have examined area-level associations of travel behaviours with obesity. One study linked the rate of obesity based on self-reported body weight and height (from the Behavioral Risk Factor Surveillance System by Center for Disease Control and Prevention) with the prevalence of active transport and car use for commuting (from the US Department of Transportation) at the state level and found that states with higher proportions of active commuters had lower proportions of obese persons^18^. Similar relationships between active travel to work and self-reported obesity at the level of cities were reported in another US study^19^. A study using travel and health survey data collected in California also found positive associations between county-level average vehicle mileage travelled and the proportion of obese individuals^20^.

These previous area-level studies have several limitations. First, they used larger geographical areas as units of analysis (i.e. state, county, and city). However, such larger areas can have considerable within-area heterogeneity in residents’ travel behaviours and health. Smaller area units such as neighbourhoods, which are likely to be more homogeneous, would be more suitable to capture between-area differences in travel behaviours and health^21^. Second, they relied on self-reported height and weight to determine body mass index as an indicator of obesity. Objectively measured waist circumference, which is a widely used indirect measure of central adiposity^22^, would produce more robust and clinically relevant evidence^23^. Another issue is related to the way travel behaviours have been aggregated at an area level, which is a key consideration in examining how within-area travel patterns are related to obesity. Two of these data-linkage studies examined the proportion of those using active modes of travel for commuting^18,19^, while Lopez-Zetina et al.^20^ focused on the area-level vehicle mileage as the exposure measure. Since what matters to people’s health is how long they engage in physically active or sedentary behaviours, it is worth categorising participants not simply based on the mode of travel but on the duration of travel behaviours. For active travel behaviours, the current public health recommendation, at least 150 min/week of moderate-intensity physical activity to obtain health benefits, is applicable^24^. There is no such guideline for the duration of car use, but an Australian study found that spending over 60 min/d in cars was associated with greater cardiometabolic risk^25^.

In addition to such duration-based metrics, it is also of interest to investigate how the ‘mix’ of travel modes is related to area-level obesity. Examining how the use of diverse transport modes in specific areas is related to obesity can be informative for transport and planning policies. It is possible to postulate that a higher level of diversity (mix of different travel modes) within an area is associated with a lower rate of obesity. It can be argued that the use of transport modes is easier to identify, compared to the proportion of residents engaging in a certain duration of active or sedentary travel. The two previous studies examining travel behaviours and obesity at an area level did use the prevalence of active travel^18,19^. Countries including Australia collect information about the main mode of travel to work in census^26^. Thus, there is a potential of using travel mode diversity as a simplified measure related to area-level obesity. However, the authors are not aware of research that examined the relationship between the diversity of travel modes and area-level obesity.

This study examined cross-sectional, neighbourhood-level associations of multiple travel behaviour metrics, which included both active and sedentary travel, with the rates of high waist circumference (based on objective measures) among Australian adults, by linking data from household travel surveys and a population health survey.

## Methods

### Household travel surveys

We used data from the 2009 South-East Queensland Travel Survey (SEQTS) and the 2009 Victorian Integrated Survey of Travel and Activity (VISTA), which had a similar format for data collection, allowing us to combine the databases. The study areas of SEQTS included Brisbane, Gold Coast, and Sunshine Coast, which had a combined population of 2.9 million in 2009. Data were collected from 27,213 participants in 10,335 households. VISTA was conducted in the Melbourne Statistical Division and surrounding regional cities (Geelong, Ballarat, Bendigo, Shepparton), which together had a population of 4.5 million in 2009. VISTA data were collected from 41,626 participants in 16,269 households. Both surveys used multistage random sampling design, in which Census Collection Districts (CCD; the smallest geographic units for Census data collection at the time of data collection) were selected first, then households were selected from each CCD. All residents in the selected households were asked to report details of each incidence of travel (origin, destination, start time, end time, mode, and purpose) on the assigned survey day using a 24-h travel diary. They also reported their socio-demographic characteristics (age, gender, employment status, household composition, income) in a self-administered questionnaire. The number of adults aged 18 years and older was 20,800 for SEQTS and 32,125 for VISTA. After excluding those who did not complete the travel diary (N = 614), and those without information on residential location (N = 13), data from 52,298 participants were analysed. These travel surveys were conducted in accordance with ethical guidelines under government statutes and regulations. Informed consent was obtained from participants.

### Population health survey

Data on obesity were collected in the 2014–15 National Health Survey, a nationally representative survey of Australians^27^. Private dwellings across urban and rural areas of Australia were randomly selected using a stratified multistage sampling design. In total, 19,259 residents participated in the survey^27^. Data were collected by trained interviewers using face-to-face interviews. Please refer to the “Outcome measure” section for details of measuring waist circumference. Data collection was conducted under the authority of the Census and Statistics Act 1905, which imposes strict confidentiality obligations.

### Unit of analysis

Data from the travel and health surveys were aggregated and linked at the level of Population Health Area (PHA). Each PHA consists of one or more Statistical Area Level 2s (SA2s). SA2s have an average population of 10,000 residents (range 3000 to 25,000) and represent a socioeconomically coherent, local community^28^. Data linkage identified 352 PHAs (138 from SEQHTS, 214 from VISTA) for analysis. They were all in urban areas since the travel survey data were collected only in major and regional cities. To ensure that each PHA had a sufficient number of travel survey participants, we employed a minimum of 30 participants, which is generally considered to be a large enough sample size for calculating estimates^29^. Of the 352 PHAs, 327 had 30+ participants. Nearly 90% of these PHAs consisted of either one (45%) or two (42%) SA2s.

### Outcome measure

In the 2014–15 National Health Survey, participants’ waist circumference was measured objectively by interviewers with participants wearing light clothes. The Public Health Information Development Unit (PHIDU)^30^ estimated the PHA-level prevalence of people aged 18+ years with a waist circumference indicating increased risk of developing chronic diseases, i.e., ≥ 94 cm for men or ≥ 80 cm for women^31^. The estimated area-level obesity rates were expressed as the age-standardised rate of high waist circumference (%) to adjust for different age distributions between PHAs.

### Exposure measures

Travel survey participants were categorised in two ways. One is duration-based category and the other is mode-based category. The former was developed according to the duration of walking/cycling and car use time. As shown in Table 1, participants were classified into active travel (AT), mixed travel (MT), sedentary travel (ST) or no travel. AT involved 1–29 min/d (low level) or 30+ min/d (high level) of walking/cycling with no car use. The cut-off of 30 min/d for walking/cycling was derived from WHO’s physical activity guidelines^24^, as the recommended minimum of 150 min/week of physical activity can be achieved by undertaking 30 min/d of walking/cycling each weekday. MT referred to those who walked/cycled and used cars on the survey day. ST involved any car use without walking/cycling. Car use included any duration of driving a car, riding a car as a passenger, and riding in a taxi, but excluded the use of motorcycles and commercial motor vehicles. Since one Australian study found car use over 60 min/d to be associated with greater cardiometabolic risk^25^, we also examined an alternative category, prolonged sedentary travel (PST), in which participants used cars over 60 min/d without walking/cycling. The use of public transport was excluded in this categorisation because its health implications are not clear due to different possible postures (sitting or standing) in public transport. We characterised each PHA by calculating the proportion of travel survey participants belonging to each of these categories.

**Table 1:** Classification of participants by the type and duration of travel behaviours.

Walking/cycling	Car use	
	0 min/d	1+ min/d
0 min/d	No travel	ST^a^
1–29 min/d	Low AT	MT
30+ min/d	High AT	

AT: active travel; MT: mixed travel; ST: sedentary travel.

^a^Sub-category: PST (prolonged sedentary travel: 0 min of walking/cycling, 60+ min/d of car use).

The mode-based category was used to identify how diverse people in a certain area are in terms of their travel modes. Participants were categorised into the following three groups based on the travel modes they used on the survey day: ‘walking/cycling only’ (those who walked or cycled without using cars or public transport), ‘car users’ (those who used cars but did not use public transport, allowing some walking/cycling), and ‘public transport users’ (those who used bus, train, tram or ferry, which normally accompanies walking/cycling). Those who used both cars and public transport (e.g. ‘park and ride’) were also categorised as public transport users. We then used the following formula to calculate the diversity of travel modes for each PHA:

$$Diversity=-\frac{\sum_{k=1}^{N}\left({p}_{k}\cdot \mathrm{ln}{p}_{k}\right)}{\mathrm{ln}N}$$

where *p* is the proportion of a mode group, and *N* is the total number of groups (= 3). The score ranges from 0 (no diversity: dominated by one travel mode) to 1 (full diversity: all modes equally represented). This is an equation for calculating ‘entropy’^32^ and has been used to measure diversity of land use^33^.

### Covariates

For each PHA, the proportion of men, older adults aged 65+ years, workers, households with children, and those of low-income households (less than AU$1100 per week) were calculated based on travel survey data. The Index of Relative Socioeconomic Disadvantage (IRSD), a composite measure of area-level deprivation^34^, was also used as a covariate. The population-weighted average of the 2011 Census IRSD scores of the SA2s included was calculated for each PHA.

### Statistical analyses

To account for spatial autocorrelation in examining the area-level associations of travel behaviour metrics with the age-standardised rate of high waist circumference, we used a Bayesian conditional autoregressive (CAR) modelling approach, with Gaussian likelihood and identity link function^35^. A set of spatial random effects were included, and an adjacency matrix *W* was used to control for the spatial autocorrelation structure of the random effects. The elements of *W* were defined by whether two PHAs shared a common border (1: two areas shared a common border, 0: no shared border). This specification enabled two adjacent areas to be autocorrelated, while the random effects were set as conditionally independent for non-adjacent areas. Estimation of model parameters for Bayesian CAR models was done using Markov Chain Monte Carlo (MCMC) simulation. Each model (see below) was first estimated for 100,000 MCMC iterations, the first 10,000 of which have been discarded as the burn-in values. The remaining 90,000 were thinned by 100 to reduce dependency between successive draws, resulting in 900 posterior estimates of regression coefficients of interest. The *Geweke* diagnostic test was used to check for the convergence of MCMC simulation. The posterior distribution of the estimated regression coefficients was then used to identify the median and 95% credible interval (2.5th, 97.5th percentiles). Statistical significance (*p* < 0.05) was assessed by observing whether zero is included within this interval.

Three sets of models were fitted for the duration-based travel behaviour categories (AT, MT and ST). Model 1 examined the association of the age-standardised rate of higher waist circumference with each metric separately. Model 2 is an extension of Model 1, adjusting for the covariates described above. Model 3 examined low AT, high AT, MT, and the proportion of those who did not travel simultaneously (leaving out ST), adjusting for the covariates. This is a substitution model, where a coefficient for low AT can be interpreted as the effect of statistically replacing one unit of low AT with the same amount of ST, since the model holds the other metrics (high AT, MT, no travel) constant, and the total of all metrics is always the same (100%). Model 3 did not involve PST, as it was a subcategory of ST. For the diversity measure, Models 1 and 2 were fitted. All regression coefficients corresponded to a 5% increment in travel behaviour metrics. All analyses were conducted in R 4.0.5 (R Core Team, Vienna, Austria). The package ‘CARBayes’ version 5.2.3 was used for Bayesian CAR models. The package ‘spdep’ version 1.1.7 was used to construct the adjacency matrix *W*.

## Results

### Characteristics of participants and study areas

After excluding 311 participants living in the PHAs with < 30 participants, 51,987 remained in the travel survey sample. Table 2 shows the characteristics of these participants. Three fifths belonged to the ST category, while the proportion was low for the AT (7% in total) and MT (11%) categories. One fifth of participants (21%) did not travel on the survey day. Regarding the mode of travel, more than 85% of participants were car users. Supplementary Table 1 shows the characteristics of travel survey participants belonging to each travel behaviour category. The mean duration of walking/cycling was less than 20 min/d for low AT and about 30 min/d for MT, while it was close to 60 min/d for high AT. The mean time spent in cars ranged from 54 to 70 min/d for MT and ST, but it was more than 100 min/d in PST. Table 3 shows the characteristics of the 327 PHAs included in the analysis. The median number of travel survey participants in these PHAs was 120 and the mean estimated rate of high waist circumference was 62% (range: 46–73%). The mean entropy value, the measure of diversity of travel modes calculated for 317 PHAs, was 0.44 (range: 0.00–0.96). Supplementary Table 2 compares the characteristics of the PHAs included and those excluded from analysis. The PHAs excluded (N = 25) were smaller in geographic size and higher in the proportion of high AT and those who did not travel, but similar in the obesity measure compared to those included (N = 327). Supplementary Table 3 shows the Pearson’s correlation coefficients between travel behaviour metrics and the outcome. The rate of high waist circumference was significantly correlated with all travel metrics, with higher coefficients observed for diversity (*r* = − 0.58), high AT (− 0.56), and ST (0.55). The diversity measure was closely correlated with the measures of high AT (0.87) and ST (− 0.92).

**Table 2:** Characteristics of 51,987 travel survey participants included in the analytical sample.

	Mean (SD) or %
Mean age (SD)	47.5 (17.1)
Gender, % men	47
Work status, % workers	67
Household with children, % yes	47
Household income, % low^a^	32
Duration-based category	
Proportion of low AT, %	2.4
Proportion of high AT, %	4.6
Proportion of MT, %	11.1
Proportion of ST, %	61.0
Proportion of PST, %	29.4
Proportion of no travel, %	20.8
Mode-based category	
Proportion of walking/cycling only^b^, %	5.0
Proportion of car users^b^, %	86.4
Proportion of public transport users^b^, %	8.6

AT: active travel; MT: mixed travel; ST: sedentary travel; PST: prolonged sedentary travel.

^a^Household income < AU$1100 pw in the travel survey.

^b^N = 41,159 after excluding those who did not travel on the survey day.

**Table 3:** Characteristics of the 327 PHAs included in the analytical sample.

	Mean (SD)	Median [95% range]
Size, km^2^	80.4 (190)	16 [3, 796]
Population count (1000 persons)^a^	20.5 (8.7)	19 [7, 38]
Population density^a^, persons/ha	14.7 (12.5)	13 [0.2, 43]
Number of travel survey participants	158 (131)	123 [36, 578]
Proportion of men, %	47.6 (3.3)	47.6 [41.3, 54.9]
Proportion of adults 65+ years, %	17.2 (8.0)	16.0 [2.4, 35.6]
Proportion of workers, %	67.9 (9.5)	68.5 [46.3, 84.5]
Proportion of households with children, %	47.2 (13.4)	47.2 [17.5, 72.3]
Proportion of low-income households^b^, %	30.0 (11.8)	29.4 [10.5, 56.1]
IRSD^c^	1020 (65)	1030 [850, 1106]
Proportion of low AT, %	2.6 (2.6)	2.0 [0.0, 9.7]
Proportion of high AT, %	5.5 (6.1)	3.7 [0.0, 24.7]
Proportion of MT, %	11.9 (6.1)	10.7 [2.6, 25.9]
Proportion of ST, %	59.2 (12.1)	60.8 [29.1, 78.1]
Proportion of PST, %	30.0 (8.8)	30.0 [14.7, 48.0]
Proportion of no travel, %	20.9 (6.7)	20.3 [9.5, 36.0]
Diversity of travel modes (entropy)^d^	0.44 (0.21)	0.41 [0.11, 0.89]
Age-standardised rate of high waist circumference, %	62.0 (5.0)	62.6 [51.5, 70.3]

AT: active travel; MT: mixed travel; ST: sedentary travel; PST: prolonged sedentary travel.

95% range: 2.5th, 97.5th percentiles.

^a^Australian Bureau of Statistics 2011 Census data.

^b^household income < AU$1100 pw in the travel survey.

^c^IRSD: Index of Relative Socio-economic Disadvantage.

^d^Diversity was calculated for 317 PHAs (the number of participants in 10 PHAs was below 30 after excluding those who did not travel on the survey day).

### Results of regression analyses

**Figure 1:**
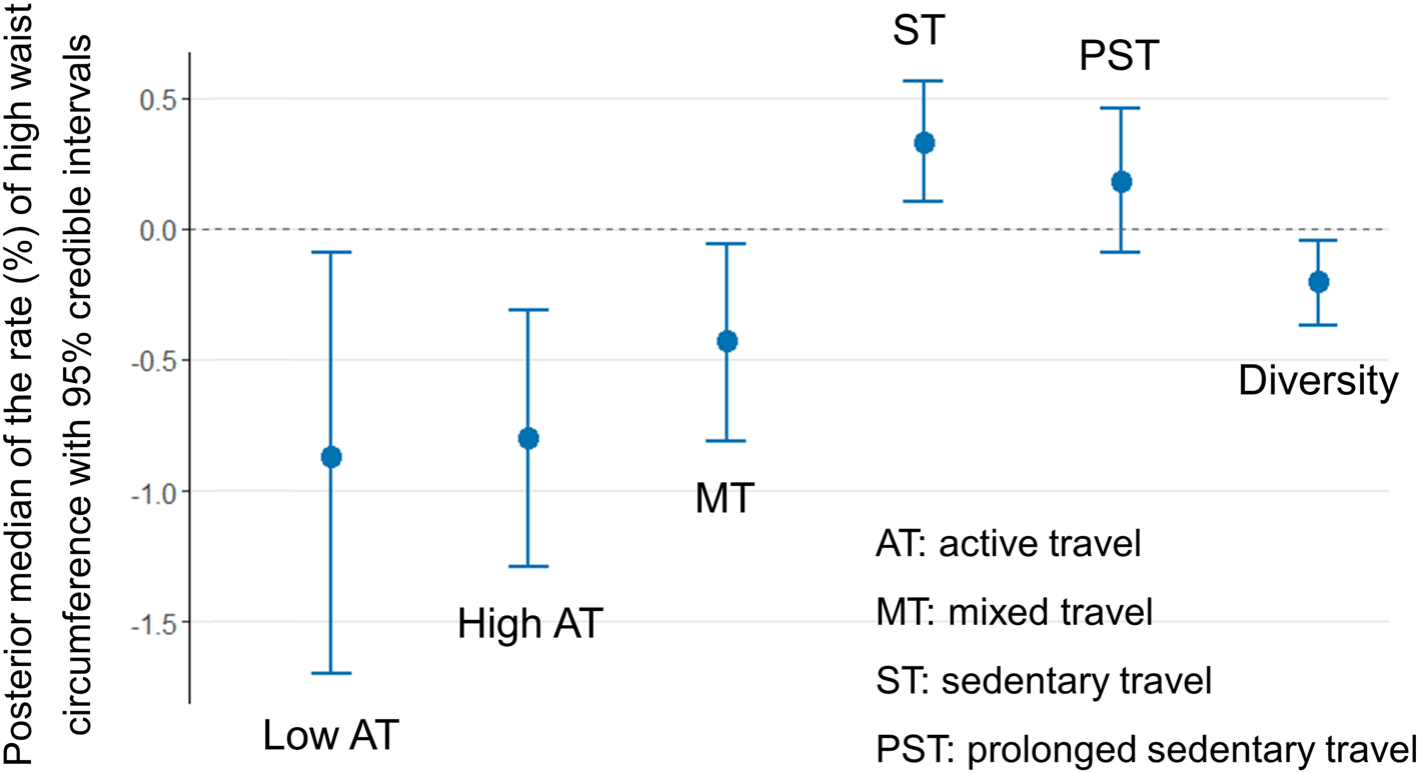
Area-level associations of the age-standardised rate (%) of high waist circumference with travel behaviour metrics. Regression coefficients (the posterior median estimated using Bayesian conditional autoregressive models) correspond to each 5% increment of the travel behaviour metric. Models examined each travel behaviour metric separately, adjusting for the covariates.

Bayesian CAR models examined associations of the age-standardised rate of higher waist circumference with travel behaviour metrics. The *Geweke* diagnostic test ensured the convergence of MCMC simulation for all regression coefficients of interest (test values are not shown, as each coefficient was estimated from a separate model). Since Model 1 (unadjusted) and Model 2 (adjusted for area-level sociodemographic covariates) produced similar regression coefficients, we presented the results of Model 2 in Fig. 1. The regression coefficients for these models are reported in Supplementary Table 4. As shown in Fig. 1, a higher proportion of low AT, high AT and MT was associated with a lower rate of high waist circumference, whereas the direction of association was opposite for ST. For instance, a 5% increment in the proportion of low AT was associated with a 0.9% lower rate of high waist circumference (Fig. 1). PST was not associated with the outcome in the model. A higher entropy value (more diversity in travel modes) was significantly associated with a lower rate of high waist circumference. Table 4 shows the results of Model 3 (substitution model), where ST was omitted from analysis. It found that statistically replacing ST with high AT was associated with a lower rate of high waist circumference. However, replacing ST with low AT was not associated with the outcome. Replacement of ST with MT approached the significance level (the 95% credible interval included the null value).

**Table 4:** Area-level associations of the age-standardised rate (%) of high waist circumference with travel behaviour metrics: substitution models.

Travel behaviour metric	Walking/cycling	Car use	Regression coefficients: Posterior median [95% credible intervals]
Low AT	1–29 min/d	0 min/d	− 0.59 [− 1.45, 0.24]
High AT	30+ min/d	0 min/d	**− 0.67 [− 1.16, − 0.18]**
MT	1+ min/d	1+ min/d	− 0.38 [− 0.77, 0.00]
No travel	0 min/d	0 min/d	− 0.05 [− 0.34, 0.25]
ST	0 min/d	1+ min/d	(substitution target)

Regression coefficients were estimated using Bayesian conditional autoregressive models, accounting for spatial autocorrelation, based on Markov Chain Monte Carlo simulation. The posterior median and 95% credible intervals of the distribution of regression coefficients correspond to each 5% increment of the travel behaviour metric. Regression coefficients shown in bold are significant at *p* < 0.05.

The model examined low AT, high AT, MT, and the proportion of those who did not travel simultaneously (ST omitted), adjusting for the proportions of men, older adults, workers, households with children, low-income households, and IRSD.

AT: active travel; MT: mixed travel; ST: sedentary travel.

## Discussion

This study examined cross-sectional associations of area-level travel behaviour metrics with the rate of high waist circumference, by linking data from household travel survey and population health survey. We found that areas where a higher proportion of participants engaged in walking/cycling had a lower rate of high waist circumference. A higher proportion of car users who did not walk/cycle was associated with a higher rate of high waist circumference. It is noticeable that the regression coefficient was greater for low AT than high AT in Models 1 and 2 (Supplementary Table 4). This may be due to covariation of the proportion of low AT and high AT (as shown in Supplementary Table 3). In other words, PHAs with a higher proportion of low AT were also higher in the proportion of high AT. The non-significant association of low AT in Model 3 (where low AT and high AT were examined simultaneously) suggests that low AT may not be independently associated with the outcome. This substitution model suggests that area-level efforts to replace ST with the higher level of AT (engaging in walking/cycling for 30+ min/d) would contribute to reduced obesity rates, but such effects may not be expected if replacement occurs between ST and the lower level of AT (1–29 min/d). A higher proportion of MT (those who walked/cycled and used a car) was also associated with a lower rate of high waist circumference in Models 1 and 2. Supplementary Table 1 shows that the mean duration of walking/cycling was over 30 min/d for participants in the MT category. It can be argued that this level of walking/cycling may mitigate the damage from car use. However, since substituting ST with this category produced a coefficient that was marginally significant (Table 4), the associations observed for MT may be partly due to its correlation with high AT. It is not clear at this stage whether area-level transport strategies simply focusing on increasing walking/cycling without reducing car use may reduce the prevalence of obesity. Our findings seem to suggest that switching car use to 30+ min/d of walking/cycling may be needed for transport strategies to be effective for obesity prevention. There is a scope for improvement in this regard. It was found that 7% of car trips in Queensland (Australia) are short enough to be walked^36^, and 18% of them in Seattle (USA) can be replaced with micromobility, which includes cycling^37^. Area-level strategies that can encourage residents to shift from car use to walking/cycling (e.g. traffic speed reduction, well-connected foot/bike paths leading to commercial areas) may help to achieve this goal.

A significant result was obtained for the diversity measure. A higher entropy, which indicates a greater diversity of travel modes, was associated with lower prevalence of high waist circumference. In the context of Australia, where car use is the dominant mode of travel^26^, a larger diversity of travel modes can be obtained by converting car users to walkers/cyclists or to public transport users. The findings suggest that a higher diversity is likely to involve a relatively high level of walking and cycling. As shown in Supplementary Table 1, those who walked/cycled (without car use or public transport use) spent on average more than 40 min/d on these active behaviours. Even public transport users engaged in over 30 min/d of walking/cycling. Our findings suggest that areas where residents can take advantage of diverse modes of travel may be conducive to lower rates of obesity. Transit-oriented development, where mixed-use residential development is clustered around public transport stops (e.g. railway stations)^38^, would provide solutions not only to traffic-related problems (traffic congestion, air pollution) but also to reduce the prevalence of obesity.

It should be also noted that the diversity score, which can be obtained only from residents’ use of different travel modes, was closely correlated with the duration-based measures of active and sedentary travel (Supplementary Table 3). The diversity measure was also more strongly correlated with the obesity rate than the other travel behaviour metrics. Our findings suggest that travel mode diversity may be employed in future research as an integrated profile of neighbourhoods related to area-level obesity.

Building on a previous individual-level study that reported associations of time spent in cars and obesity-related measures^25^, we examined the proportion of those who used cars ≥ 60 min/d without walking/cycling (PST). Contrary to the expectation, we did not find any association between this category and the outcome. Supplementary Table 3 shows that the correlation coefficient between PST and the outcome was 0.29, which was lower than the correlation with ST (0.55). It is difficult to give a plausible explanation about why this is the case. Supplementary Table 1 shows that participants in the PST category were slightly younger, more likely to be working, and less likely to be in low-income households, compared to those in the ST category. Unmeasured area-level confounders such as dietary patterns and recreational physical activities may have contributed to the unexpected finding.

There are a few area-level studies on travel behaviours and obesity measures. Our findings are consistent with what these previous studies have reported: the beneficial association of active commuting (state- or city-level) and the deleterious association of county-level vehicle mileage with self-reported measures of obesity^18–20^. However, our study found similar associations using a smaller area unit representing neighbourhoods, suggesting that the spatial distribution of obesity that exists at the neighbourhood level can be attributable to some extent to residents’ travel behaviour patterns. This further suggests that it is possible to address the local prevalence of obesity by developing and implementing neighbourhood-level planning initiatives. Strategies discussed above (e.g., pedestrian infrastructure, transit-oriented development) could enable residents to shift from car use to walking/cycling for daily travel. Future research is warranted to identify specific environmental characteristics or transport policies that can facilitate reduction of car dependency. Obesity prevention planning efforts may be more effective by targeting neighbourhoods that have a high prevalence of obesity and high reliance on cars for travel. Public health and planning sectors need to work together to implement such initiatives.

This is the first Australian study in which travel and health survey data have been linked to understand the area-level associations of travel behaviours and obesity. The strengths of the study include that we examined multiple area-level travel behaviour metrics, those considering the duration of walking/cycling and car use (with known health implications) and the mix of travel modes. The outcome of the study was based on measured waist circumference, which was standardised to correct for the different age distribution between the study areas. We also used a rigorous analysis technique to account for spatial autocorrelation between neighbouring PHAs.

The study limitations include temporal mismatch of travel and health surveys. Two travel surveys were conducted in 2009, while health survey data were collected in 2014–15. However, this issue may not be critical, as travel behaviours tend to be stable over time^39^, and the outcome was measured after travel survey data collection. Longitudinal studies examining area-level associations between the change in travel behaviours and the change in obesity rates would help to further improve the understanding of the role of travel behaviours in obesity prevention. The area unit used was PHA (Public Health Area) because the national health survey data were aggregated at this level. This is not a commonly used geographical unit for transport planning. However, most PHAs consisted of one or two SA2s, each of which approximately represents a local community. We focused on the relationships between area-level measures of travel behaviours and obesity. The results, expressed in regression coefficients (shown in Fig. 1 and Supplementary Table 4), may seem abstract. To inform those involved in planning of the health implications of travel behaviours, future research needs to illustrate how areas with different travel profiles vary in residents’ mean waist circumference (or other obesity related measures). Finally, the findings are likely to be generalisable to localities where car use is the dominant mode of travel but may not apply to areas where travel behaviour patterns are different. For instance, in high-density urban centres where car use is restricted or inconvenient, a lower diversity of travel modes (i.e. most residents walk/cycle for daily travel) can be associated with reduced health risk.

In conclusion, our findings support the relevance of active travel and car dependency to obesity outcomes, which is consistent with the previous studies and reviews on the same topic^9–15^. However, we also produced novel findings with implications for obesity prevention by integrating active and sedentary travel measures within the study. First, area-level initiatives encouraging a shift from car use to active travel (30+ min/d of walking/cycling) may be effective in lowering obesity rates. Second, increasing the diversity of travel modes (providing more options for travel) within an area may reduce the prevalence of obesity, suggesting that travel mode diversity can be used as an integrated travel profile index for a neighbourhood. Transport planning strategies that can facilitate such community-level changes in travel patterns would include the development of infrastructure for active travel such as walking and cycling trails linking residential areas to commercial centres. Decentralising employment opportunities, which could increase the proportion of people using active modes of travel due to a shorter distance to work, is another strategy. Since such interventions contribute not only to human health but also to environmental health through lower greenhouse gas emissions and fewer resources required for automobile infrastructure, joint obesity prevention efforts between transport, planning and public health sectors are likely to generate multiple benefits contributing to the UN’s Sustainable Development Goals.

## Supplementary Information

Supplementary Tables.

## Data Availability

Data used for the study are available from the following websites. SEQTS: https://www.data.qld.gov.au/dataset/2009-south-east-queensland-household-travel-survey. VISTA: https://transport.vic.gov.au/about/data-and-research/vista. Waist circumference data: https://phidu.torrens.edu.au/notes-on-the-data/health-status-disability-deaths/est-waist-circumference.
