# Supplementary material for: Area-level associations of travel behaviour metrics with waist circumference: findings from linkage of travel and health surveys
Source: Sci Rep. 2023 May 19;13:8136. doi: 10.1038/s41598-023-35335-w (PMC10199009; doi:10.1038/s41598-023-35335-w)
Supplement: Supplementary file 1 — Supplementary Tables. [file 41598_2023_35335_MOESM1_ESM.docx]

**Supplementary Table 1.** Characteristics of travel survey participants belonging to each travel behaviour category

|  | Duration-based category | | | | | |  | Mode-based category | | |
| --- | --- | --- | --- | --- | --- | --- | --- | --- | --- | --- |
|  | Low AT ^b^ | High AT ^b^ | MT ^b^ | ST ^b^ | PST ^b^ | No travel ^b^ |  | Walking  cycling only ^c^ | Car users ^c^ | Public transport users ^c^ |
| Proportion, % | 2.4 | 4.6 | 11.1 | 61.0 | 29.4 | 20.9 |  | 5.0 | 86.4 | 8.6 |
| Age, mean (SD) | 47.1 (19.6) | 44.3 (17.8) | 44.6 (16.0) | 47.0 (15.9) | 45.5 (14.8) | 51.5 (19.8) |  | 48.6 (18.4) | 47.0 (15.9) | 40.1 (16.7) |
| Gender, % men | 46 | 50 | 43 | 49 | 51 | 46 |  | 50 | 48 | 45 |
| Proportion of workers, % | 55 | 62 | 73 | 73 | 80 | 47 |  | 53 | 73 | 76 |
| Proportion of households with children, % | 35 | 35 | 48 | 51 | 54 | 39 |  | 35 | 50 | 43 |
| Proportion of low-income households,^a^ % | 46 | 37 | 24 | 28 | 23 | 44 |  | 43 | 28 | 27 |
| Mean duration of walking/ cycling (SD), min/d | 17.8 (6.5) | 56.8 (30.7) | 31.4 (29.7) | 0 (0) | 0 (0) | 0 (0) |  | 44.3 (36.0) | 3.6 (14.9) | 34.2 (23.4) |
| Mean duration of car use (SD), min/d | 0 (0) | 0 (0) | 54.3 (47.7) | 69.5 (58.0) | 110 (59.3) | 0 (0) |  | 0 (0) | 68.7 (57.3) | 21.3 (33.6) |
| Mean duration of public transport use (SD), min/d | 21.7 (34.9) | 26.7 (40.8) | 22.7 (42.3) | 0.11 (5.88) | 0.19 (8.10) | 0 (0) |  | 0 (0) | 0 (0) | 63.7 (47.0) |

^a^ Household income < AU$1100 pw in the travel survey

^b^ N=51,987

^c^ N=41,159 after excluding those who did not travel on the survey day

AT: active travel; MT: mixed travel; ST: sedentary travel; PST: prolonged sedentary travel

**Supplementary Table 2.** Characteristics of PHAs included in and excluded from analyses

|  | Mean (SD) or Median [Q1, Q3] | |
| --- | --- | --- |
|  | Included in analysis | Excluded analysis |
| Number of PHAs | 327 | 25 |
| Number of participants | 51,987 | 311 |
| Size, km^2^ | 16 [9, 54] | 9 [5, 43] |
| Population count (1000 persons) ^a^ | 19 [14, 27] | 14 [10, 20] |
| Population density,^a^ persons/ha | 13 [4, 21] | 13 [1, 23] |
| Number of participants per PHA | 120 [73, 200] | 18 [13, 24] |
| Proportion of low AT, % | 2.6 (2.6) | 2.3 (3.2) |
| Proportion of high AT, % | 5.5 (6.1) | 9.0 (22.2) |
| Proportion of MT, % | 11.9 (6.1) | 7.8 (9.4) |
| Proportion of ST, % | 59.2 (12.1) | 53.8 (27.3) |
| Proportion of PST, % | 30.0 (8.8) | 28.7 (20.3) |
| Proportion of no travel, % | 20.9 (6.7) | 27.2 (26.7) |
| Diversity of travel modes (entropy) ^b^ | 0.44 (0.21) | 0.35 (0.30) |
| Age-standardised rate of high waist circumference, % | 62.0 (5.0) | 62.7 (6.6) |

^a^ Australian Bureau of Statistics 2011 Census data

^b^ The number of PHAs included in analysis was 317, and those excluded was 35.

AT: active travel; MT: mixed travel; ST: sedentary travel; PST: prolonged sedentary travel

**Supplementary Table 3.** Pearson’s correlation coefficients between travel behaviour metrics and the outcome measure

|  | Low AT | High AT | MT | ST | PST | Diversity |
| --- | --- | --- | --- | --- | --- | --- |
| Low AT | — |  |  |  |  |  |
| High AT | 0.64 | — |  |  |  |  |
| MT | 0.47 | 0.57 | — |  |  |  |
| ST | -0.68 | -0.81 | -0.66 | — |  |  |
| PST | -0.45 | -0.53 | -0.40 | 0.71 | — |  |
| Diversity | 0.73 | 0.87 | 0.70 | -0.92 | -0.63 | — |
| Age-standardised rate of high waist circumference | -0.47 | -0.56 | -0.52 | 0.55 | 0.29 | -0.58 |

All coefficients at *p* < .001

Correlation was calculated for 327 PHAs, except for those involving diversity (317 PHAs)

AT: active travel; MT: mixed travel; ST: sedentary travel; PST: prolonged sedentary travel

**Supplementary Table 4.** Area-level associations of the age-standardised rate (%) of high waist circumference with travel behaviour metrics

| Travel behaviour metric | Walking/  cycling | Car use | Regression coefficients  Posterior median [95% credible intervals] | |
| --- | --- | --- | --- | --- |
|  |  |  | Model 1 | Model 2 |
| Low AT | 1–29 min/d | 0 min/d | **-1.00 [-1.85, -0.14]** | **-0.87 [-1.70, -0.09]** |
| High AT | 30+ min/d | 0 min/d | **-0.80 [-1.23, -0.37]** | **-0.80 [-1.29, -0.31]** |
| MT | 1+ min/d | 1+ min/d | **-0.45 [-0.81, -0.07]** | **-0.43 [-0.81, -0.06]** |
| ST | 0 min/d | 1+ min/d | **0.31 [0.11, 0.52]** | **0.33 [0.10, 0.56]** |
| PST | 0 min/d | 60+ min/d | 0.08 [-0.17, 0.32] | 0.18 [-0.09, 0.46] |
| Diversity | — | — | **-0.17 [-0.32, -0.03]** | **-0.20 [-0.37, -0.05]** |

Regression coefficients were estimated using Bayesian conditional autoregressive models, accounting for spatial autocorrelation, based on Markov Chain Monte Carlo simulation. The posterior median and 95% credible intervals of the distribution of regression coefficients correspond to each 5% increment of the travel behaviour metric. Regression coefficients shown in **bold** are significant at *p* < .05.

Model 1 examined each travel behaviour metric in separate models (unadjusted)

Model 2 examined each travel behaviour metric in separate models, adjusting for covariates (proportions of men, older adults, workers, households with children, low-income households, and IRSD).

AT: active travel; MT: mixed travel; ST: sedentary travel; PST: prolonged sedentary travel
